# Supplementary material for: Analysis of Aroma Characteristics of ‘Binzi’ and ‘Xiangguo’ Apple—Ancient Cultivars in China
Source: Foods. 2024 Sep 10;13(18):2869. doi: 10.3390/foods13182869 (PMC11431139; doi:10.3390/foods13182869)
Supplement: Supplementary file 1 [file foods-13-02869-s001.zip › foods-3181072-supplementary.pdf]

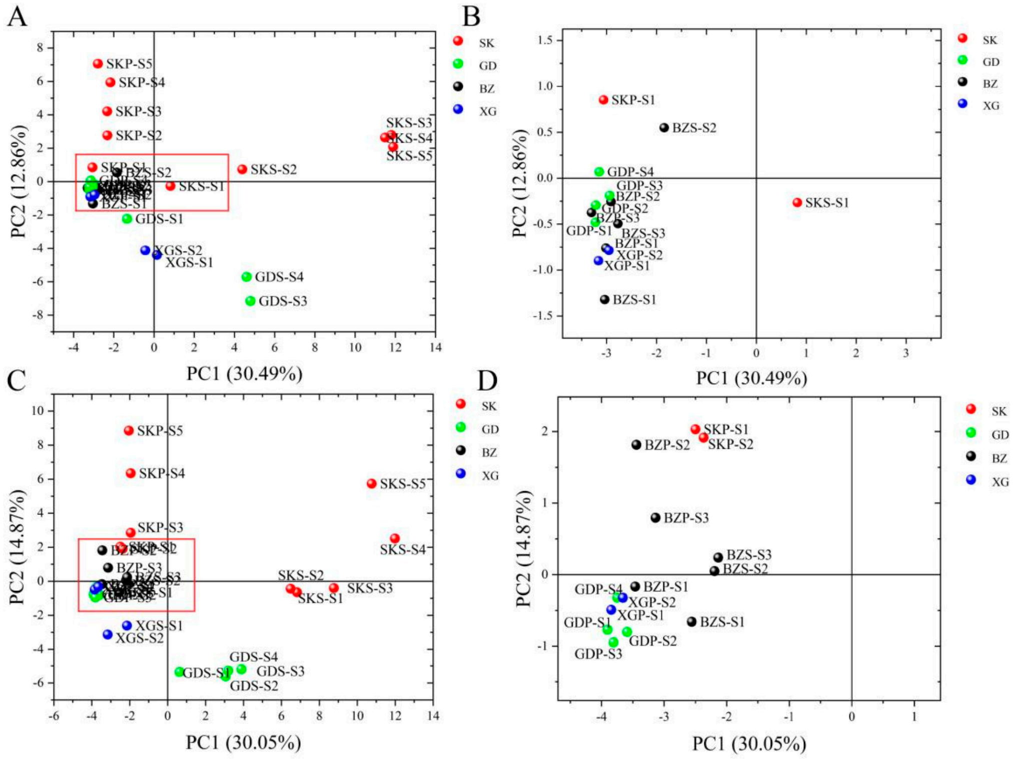

Figure S1. PCA of four apple cultivars in the first year (A) and second year (C). (B) and (D) are partial enlarged images within the red border in (A) and (C), respectively. SKS, GDS, BZS, XGS, and XGS represent skin samples from four apple cultivars, respectively. SKP, GDP, BZP, XGP, and XGP represent pulp samples from four apple cultivars, respectively. The same applies below.

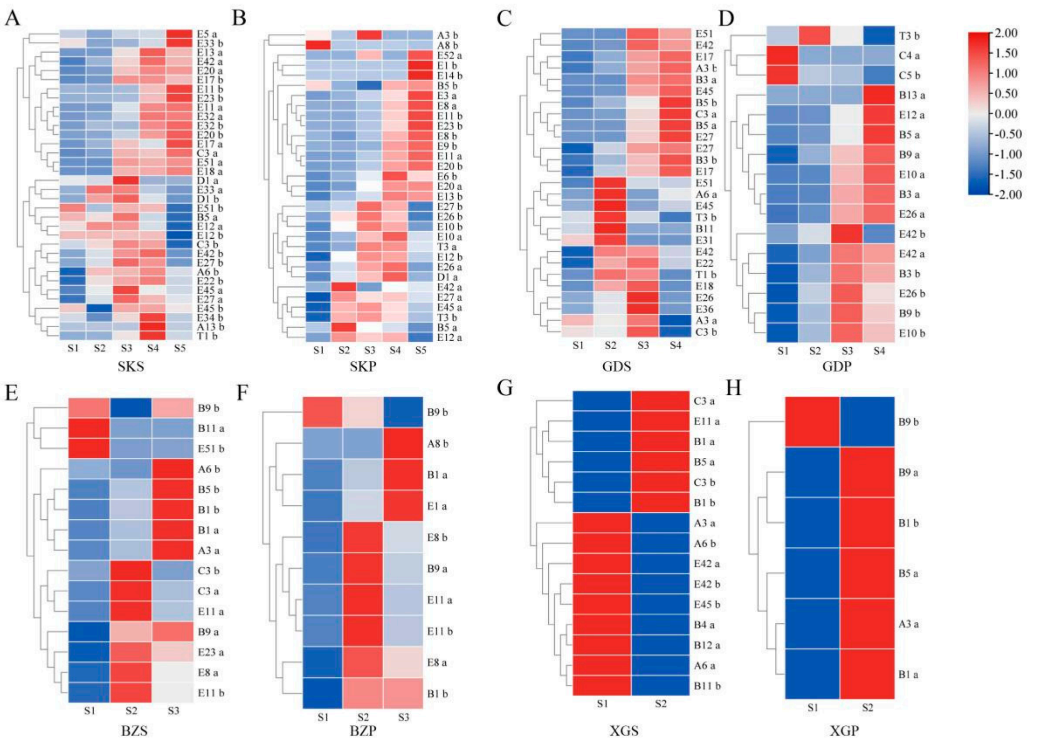

Figure S2. Heatmap of D-VOCs among four apple cultivars. The codes of VOCs are shown in Table S1. The letters 'a' and 'b' represent the first year and second year, respectively.

Table S1. Detection results and aroma description of VOCs by GC-MS.

Class	Code	CAS	Compound	Detected in				Aroma Description	Odor threshold（μg/kg）
				skin at 2017	pulp at 2017	skin at 2018	pulp at 2018		
Esters	E1	141-78-6	Ethyl acetate	+	+	+	+	Pineapple, balsamic	750
	E2	105-37-3	Ethyl propionate	–	+	–	–	Banana, apple	10
	E3	592-84-7	Butyl formate	+	+	+	+	Fruit	n.f.
	E4	5145-01-7	2-Methyl-4-pentanolide	+	–	–	–	n.f.	n.f.
	E5	868-57-5	Methyl 2-methylbutyrate	+	–	+	–	Apple, Fruit, Green Apple, Strawberry	0.25
	E6	109-60-4	Propyl acetate	–	+	+	+	Celery，floral, pear, fruity	2700
	E7	110-19-0	Isobutyl acetate	–	+	–	+	Fruit, apple, banana, sweet	66
	E8	105-54-4	Ethyl butyrate	+	+	+	+	Pineapple, fruity, apple	1
	E9	106-36-5	Propyl propionate	–	+	+	+	Apple, Banana, Pineapple	57
	E10	123-86-4	Butyl acetate	+	+	+	+	Apple, Banana	66
	E11	7452-79-1	Ethyl 2-methylbutyrate	+	+	+	+	Apple, Ester, Green Apple, Kiwi, Strawberry	0.1
	E12	624-41-9	2-Methylbutyl acetate	+	+	+	+	Apple, Banana, Pear	5
	E13	105-66-8	Propyl butyrate	+	+	+	+	Pineapple, solvent	18
	E14	539-82-2	Ethyl valerate	–	+	+	+	Yeast, fruit	5.8
	E15	590-01-2	Butyl propionate	–	–	+	+	Red Fruit, Strawberry	25
	E16	628-63-7	Amyl acetate	–	–	–	+	Apple, Banana, Pear	2
	E17	629-33-4	Hexyl formate	+	–	+	–	Fruit	n.f.
	E18	106-70-7	Methyl hexanoate	+	+	+	+	Apple, fruity, green, sweet, unripe	70

	E19	1191-16-8	Prenyl acetate	–	+	–	–	n.f.	n.f.
	E20	37064-20-3	Propyl 2-methylbutyrate	+	+	+	+	n.f.	n.f.
	E21	105-68-0	Isoamyl propionate	+	+	+	+	Apple, Apricot, Pineapple	n.f.
	E22	109-21-7	Butyl butyrate	+	+	+	+	Banana	100
	E23	123-66-0	Ethyl caproate	+	+	+	+	Apple peel, fruit	1
	E24	35608-64-1	Ethyl 3-Hydroxybutyrate	+	+	–	–	n.f.	n.f.
	E25	2396-84-1	Ethyl sorbate	–	+	–	+	Fruit	n.f.
	E26	142-92-7	Hexyl acetate	+	+	+	+	Apple, Banana, Grass	2
	E27	15706-73-7	2-Methyl-butanoic acid butyl ester	+	+	+	+	Banana	17
	E28	61692-83-9	Propyl tiglate	–	–	+	+	n.f.	n.f.
	E29	51115-64-1	2-Methylbutylbutyrate	+	+	–	+	n.f.	n.f.
	E30	106-27-4	Isoamyl butyrate	–	–	+	–	Fruit	0.13
	E31	540-18-1	Amyl butyrate	+	–	+	–	Banana	210
	E32	626-77-7	Propyl hexanoate	+	+	+	+	Berry, Fruit, Petrol, Pineapple	n.f.
	E33	2445-78-5	2-Methylbutyl 2-methylbutyrate	+	+	+	+	Apple, Berry, Rum	n.f.
	E34	2445-76-3	Hexyl propionate	+	+	+	+	Sweet, fruit	8
	E35	112-32-3	Octyl formate	–	+	–	+	Floral	n.f.
	E36	111-11-5	Methyl octanoate	+	–	+	–	Fruit, Orange, Wax, Wine	200
	E37	2349-07-7	Hexyl isobutyrate	–	–	+	–	Fruit	6
	E38	68039-26-9	Amyl 2-methyl butyrate	–	–	+	–	n.f.	n.f.
	E39	2639-63-6	Hexyl butyrate	+	–	+	–	Apple peel	250
	E40	105-79-3	Isobutyl hexanoate	–	–	+	–	Green, Spice	n.f.
	E41	55590-83-5	2-Methylbutylpentanoate	–	–	+	–	n.f.	n.f.
	E42	626-82-4	Butyl hexanoate	+	+	+	+	Fruit, Grass, Green	700
	E43	106-32-1	Ethyl caprylate	+	–	+	–	Apple, fruity, sweet	5
	E44	1731-84-6	Nonanoic acid, methylester	+	–	+	–	Coconut, Floral, Fruit	n.f.
	E45	10032-15-2	Hexyl 2-methylbutyrate	+	+	+	+	Fruity, green, apple	6
	E46	2601-13-0	2-Methylbutyl caproate	+	–	+	–	n.f.	n.f.
	E47	2351-90-8	2-Octenoic acid, ethyl ester	+	–	+	–	n.f.	n.f.
	E48	540-07-8	Hexanoic acid, pentyl ester	+	–	+	–	Banana, pineapple	4.38
	E49	624-13-5	Octanoic acid, propyl ester	+	–	+	–	n.f.	n.f.
	E50	61692-84-0	2-Methyl-, 2-methylpropyl este	+	–	+	–	n.f.	n.f.
	E51	6378-65-0	Hexyl hexanoate	+	–	+	+	Fresh fruit	10
	E52	2051-50-5	1-Methylheptyl acetate	–	+	–	+	n.f.	n.f.
	E53	3025-30-7	Ethyl-2-trans-4-cis-decadienoate	+	–	+	–	Pear	n.f.
	E54	76649-16-6	Ethyl trans-4-decenoate	–	–	+	–	Fruit	n.f.
	E55	1117-55-1	hexyl caprylate	+	–	+	–	Vegetable, fruit	2
Aldehydes	A1	96-17-3	2-Methylbutyraldehyde	+	+	+	–	Almond, Cocoa, Fermented, Hazelnut, Malt	1
	A2	110-62-3	Pentanal	+	+	+	–	Banana, grass, lower aldehyde	12
	A3	66-25-1	Hexanal	+	+	+	+	Grass, green, leaves, vinous	4
	A4	6728-26-3	trans-2-Hexenal	+	–	+	–	Almond, bitter, green	17
	A5	111-71-7	Heptaldehyde	+	+	+	–	Fresh, herbal	3
	A6	57266-86-1	(Z)-Hept-2-en	+	+	+	+	Fatty, fruity, green	13
	A7	4313-03-5	Trans,trans-2,4-heptadienal	+	+	+	+	Nut, fat	n.f.
	A8	2548-87-0	(E)-2-Octenal	+	+	+	+	Sweet, green, fatty, herbal, cucumber	90
	A9	15764-16-6	2,4-Dimethylbenzaldehyde	–	+	–	+	Fruit	n.f.

	A10	124-19-6	1-Nonanal	+	–	+	–	Aldehyde, citrus, fatty, floral, green	1
	A11	5910-87-2	(E,E)-2,4-Nonadienal	+	–	–	–	Cereal, Deep Fried, Fat, Watermelon	0.09
	A12	3913-81-3	Trans-2-decenal	+	–	+	+	Fatty, mushroom	10
	A13	5392-40-5	Citral	+	–	+	+	Lemon	32
	A14	2363-88-4	2,4-Decadienal	+	–	+	–	Aldehyde, fatty, oily, wax	0.07
	A15	2463-77-6	2-Undecenal	+	–	+	+	sweet	n.f.
	A16	2497-25-8	2-Decenal, (2Z)-	–	–	+	+	n.f.	n.f.
Alcohols	B1	64-17-5	Ethanol	+	+	+	+	Alcoholic, strong	620
	B2	78-83-1	2-Methyl-1-propanol	+	+	+	+	n.f.	n.f.
	B3	71-36-3	1-Butanol	+	+	+	+	Floral, fragrant, fruity, sweet	500
	B4	616-25-1	1-Penten-3-ol	+	+	+	+	Burnt, butter, grass, green	400
	B5	34713-94-5	2-Methyl-1-butanol	+	+	+	+	Alcoholic, banana, iodoform, vinous	250
	B6	71-41-0	1-Pentanol	+	–	+	+	Fruit，alcoholic, sweet	800
	B7	513-85-9	2,3-Butanediol	–	+	+	–	Fruity	n.f.
	B8	626-89-1	4-Methyl-1-pentanol	–	+	–	–	n.f.	n.f.
	B9	111-27-3	Hexyl alcohol	+	+	+	+	Resin, flower, green	500
	B10	928-94-9	2-Hexen-1-ol, (2Z)-	–	+	–	+	Leaf, green, wine, fruit	359.3
	B11	928-95-0	(E)-2-Hexen-1-ol	+	+	+	+	n.f.	n.f.
	B12	3391-86-4	1-Octen-3-ol	+	+	+	+	Fatty, fruity, grass, mushroom, sweet	10
	B13	23433-05-8	1,3-Octanediol	+	+	–	+	n.f.	n.f.
Ketones	C1	565-69-5	2-Methyl-3-pentanone	+	–	+	–	n.f.	n.f.
	C2	2918-13-0	1-Hepten-3-one	–	–	+	+	n.f.	n.f.
	C3	110-93-0	6-Methyl-5-hepten-2-one	+	+	+	+	Citrus Pepper, ushroom,rubber	50
	C4	3796-70-1	Geranyl acetone	+	+	+	+	Fruit	60
	C5	14901-07-6	β-Ionone	–	+	–	+	Seaweed, violet, flower, raspberry	7
	C6	23726-93-4	β-Damascenone	–	+	+	+	Apple. rose, honey	0.05
	C7	16647-04-4	6-Methyl-3,5-hepten-2-one	+	–	+	–	n.f.	n.f.
Acids	D1	116-53-0	2-Methyl butyric acid	+	+	+	+	Pungent, cheese, fruity	5.8
	D2	142-62-1	Hexanoic acid	+	–	+	–	n.f.	n.f.
	D3	63169-61-9	2-Methylpentanoic anhydride	+	–	+	–	n.f.	n.f.
Terpenes	T1	26560-14-5	α-Farnesene	+	+	+	+	Wood, sweet	n.f.
	T2	78-70-6	Linalool	+	–	+	–	flower, lavender	6
	T3	140-67-0	Estragole	+	+	+	+	Fennel	n.f.
Others	O1	110-54-3	Hexane	+	+	–	–	n.f.	n.f.
	O2	1002-33-1	1,3-Octadiene	+	–	–	–	n.f.	n.f.
	O4	54833-23-7	10-Methyleicosane	–	+	–	+	n.f.	n.f.
	O5	544-76-3	Hexadecane	+	–	+	+	n.f.	n.f.
	O6	111-67-1	2-Octene	+	–	–	–	n.f.	n.f.
	O7	629-78-7	Heptadecane	+	–	+	–	n.f.	n.f.
	O8	998-94-7	4-Methyl-1,5-heptadiene	+	–	+	–	n.f.	n.f.
	O9	4229-91-8	2-Propylfuran	+	–	+	–	n.f.	n.f.

n.f.: not found; +: detected; –: not detected.

Table S2. The content of D-VOCs in the skin of four apple cultivars (μg/kg).

Code	Compound	Starking										Golden Delicious								Binzi						Xianggguo			
		1st year					2nd year					1st year				2nd year				1st year			2nd year			1st year		2nd year	
		S1	S2	S3	S4	S5	S1	S2	S3	S4	S5	S1	S2	S3	S4	S1	S2	S3	S4	S1	S2	S3	S1	S2	S3	S1	S2	S1	S2
E5	Methyl 2-methylbutyrate	347.09	0.00	419.84	452.61	1966.79	-	-	-	-	-	-	-	-	-	-	-	-	-	-	-	-	-	-	-	-	-	-	-
E8	Ethyl butyrate	-	-	-	-	-	-	-	-	-	-	-	-	-	-	-	-	-	-	0.00	3439.60	1727.43	-	-	-	-	-	-	-
E11	Ethyl 2-methylbutyrate	33.21	56.29	926.87	2112.17	2335.16	143.97	87.36	207.78	993.16	1867.73	-	-	-	-	-	-	-	-	0.00	1546.96	383.70	227.76	1265.17	743.81	60.83	874.04	-	-
E12	2-Methylbutyl acetate	770.88	1070.98	1022.30	628.30	42.27	801.16	798.47	788.57	792.93	293.14	-	-	-	-	-	-	-	-	-	-	-	-	-	-	-	-	-	-
E13	Propyl butyrate	26.98	40.14	496.77	989.11	648.25	-	-	-	-	-	-	-	-	-	-	-	-	-	-	-	-	-	-	-	-	-	-	-
E17	Hexyl formate	521.54	602.59	845.18	718.71	1091.56	363.51	396.70	785.29	789.45	703.44	500.59	772.53	1438.34	1661.06	482.06	798.66	1025.49	1262.68	-	-	-	-	-	-	-	-	-	-
E18	Methyl hexanoate	0.00	0.00	1072.31	1119.67	1368.75	-	-	-	-	-	-	-	-	-	0.00	244.43	347.50	0.00	-	-	-	-	-	-	-	-	-	-
E20	Propyl 2-methylbutyrate	88.19	344.16	1660.23	2202.62	2043.05	427.98	488.13	910.37	1859.78	2340.42	-	-	-	-	-	-	-	-	-	-	-	-	-	-	-	-	-	-
E22	Butyl butyrate	-	-	-	-	-	420.23	712.66	805.14	867.97	636.59	-	-	-	-	440.16	779.34	861.92	631.01	-	-	-	-	-	-	-	-	-	-
E23	Ethyl caproate	-	-	-	-	-	118.34	156.73	223.12	900.67	1644.81	-	-	-	-	-	-	-	-	0.00	1167.97	782.60	-	-	-	-	-	-	-
E26	Hexyl acetate	-	-	-	-	-	-	-	-	-	-	329.83	533.10	1065.67	280.20	-	-	-	-	-	-	-	-	-	-	-	-	-	-
E27	2-Methyl-butanoic acid butyl ester	0.00	891.66	1902.59	1365.68	939.37	951.55	1116.10	1477.24	1376.66	905.80	0.00	0.00	478.95	940.38	310.83	500.95	650.18	694.77	-	-	-	-	-	-	-	-	-	-
E31	Amyl butyrate	-	-	-	-	-	-	-	-	-	-	-	-	-	-	74.35	147.60	0.00	0.00	-	-	-	-	-	-	-	-	-	-
E32	Propyl hexanoate	110.25	220.90	584.95	1475.65	1441.60	448.42	719.58	686.16	1777.86	1998.51	-	-	-	-	-	-	-	-	-	-	-	-	-	-	-	-	-	-
E33	2-Methylbutyl 2-methylbutyrate	187.57	674.94	629.79	270.98	119.51	671.76	496.73	526.94	606.41	943.97	-	-	-	-	-	-	-	-	-	-	-	-	-	-	-	-	-	-

E34	Hexyl propionate	-	-	-	-	-	402.03	285.79	455.61	632.10	381.18	-	-	-	-	-	-	-	-	-	-	-	-	-	-	-	-	-	-
E36	Methyl octanoate	-	-	-	-	-	-	-	-	-	-	-	-	-	-	117.19	150.62	239.09	107.72	-	-	-	-	-	-	-	-	-	-
E42	Butyl hexanoate	585.16	892.23	1344.35	1804.05	1483.21	1573.70	1762.84	2039.76	2151.59	1518.84	189.42	292.08	1476.77	1206.39	1209.69	1892.14	1841.24	1554.51	-	-	-	-	-	-	768.17	426.35	122.92	0.00
E45	Hexyl 2-methylbutyrate	623.44	1719.78	3162.51	1769.40	1529.23	2214.37	1653.39	2294.47	2303.84	2140.56	77.81	149.83	1103.35	1453.97	1046.85	1213.82	1039.18	1074.06	-	-	-	-	-	-	-	-	98.75	0.00
E51	Hexyl hexanoate	0.00	0.00	1161.39	1052.28	1226.00	1499.21	1182.04	1401.56	1388.55	988.17	0.00	0.00	1247.48	923.33	1030.52	1368.27	1148.10	1136.03	-	-	-	513.29	0.00	8.77	-	-	-	-
A3	Hexanal	-	-	-	-	-	-	-	-	-	-	1282.79	1030.21	1631.37	0.00	139.47	236.47	472.48	562.64	93.26	171.20	454.31	-	-	-	1570.56	1028.87	-	-
A6	(Z)-Hept-2-en	-	-	-	-	-	523.02	654.68	659.54	685.07	615.04	0.00	302.85	0.00	98.01	-	-	-	-	-	-	-	78.77	49.38	357.97	218.69	0.00	121.79	18.47
A13	Citral	-	-	-	-	-	46.65	52.94	56.86	1026.04	88.78	-	-	-	-	-	-	-	-	-	-	-	-	-	-	-	-	-	-
B1	Ethanol	-	-	-	-	-	-	-	-	-	-	-	-	-	-	-	-	-	-	0.00	233.95	973.26	0.00	173.18	623.53	0.00	501.49	0.00	233.21
B3	1-Butanol	-	-	-	-	-	-	-	-	-	-	0.00	0.00	1414.97	1623.72	164.06	448.47	834.64	1189.89	-	-	-	-	-	-	-	-	-	-
B4	1-Penten-3-ol	-	-	-	-	-	-	-	-	-	-	-	-	-	-	-	-	-	-	-	-	-	-	-	-	252.86	0.00	-	-
B5	2-Methyl-1-butanol	1640.31	1464.97	1884.47	1298.45	763.74	-	-	-	-	-	0.00	0.00	430.66	901.97	59.74	128.50	209.76	404.97	-	-	-	159.79	335.89	813.38	151.81	521.82	-	-
B9	Hexyl alcohol	-	-	-	-	-	-	-	-	-	-	-	-	-	-	-	-	-	-	379.26	1486.10	1772.29	842.42	539.24	794.19	-	-	-	-
B11	(E)-2-Hexen-1-ol	-	-	-	-	-	-	-	-	-	-	108.06	509.43	0.00	0.00	-	-	-	-	523.72	0.00	0.00	-	-	-	-	-	261.98	44.92
B12	1-Octen-3-ol	-	-	-	-	-	-	-	-	-	-	-	-	-	-	-	-	-	-	-	-	-	-	-	-	226.82	0.00	-	-
C3	6-Methyl-5-hepten-2-one	744.95	1137.22	3234.37	2981.60	4143.94	1677.90	1862.41	2144.08	2053.67	1151.50	244.07	376.62	1459.36	2809.78	1610.47	1562.29	1939.99	1127.85	228.80	3674.34	973.51	1031.23	1431.05	1025.47	1520.48	2641.47	1359.52	1675.18
D1	2-Methyl butyric acid	963.88	723.05	3517.33	0.00	0.00	864.55	1813.62	2189.72	1277.32	719.69	-	-	-	-	219.93	406.13	216.25	102.03	-	-	-	-	-	-	-	-	-	-
T1	α-Farnesene	-	-	-	-	-	115.58	123.88	284.86	597.18	211.76	-	-	-	-	252.77	708.02	659.62	242.14	-	-	-	-	-	-	-	-	-	-

-: no significant difference.

Table S3. The content of D-VOCs in the pulp of four apple cultivars (μg/kg).

VOCs	Compound	Starking										Golden Delicious								Binzi						Xiangguo			
		1st year					2nd year					1st year				2nd year				1st year			2nd year			1st year		2nd year	
		S1	S2	S3	S4	S5	S1	S2	S3	S4	S5	S1	S2	S3	S4	S1	S2	S3	S4										
E1	Ethyl acetate	-	-	-	-	-	0.00	0.00	0.00	0.00	182.21	-	-	-	-	-	-	-	-	0.00	155.92	428.07	-	-	-	-	-	-	-
E3	Butyl formate	0.00	66.82	164.30	367.25	704.76	-	-	-	-	-	-	-	-	-	-	-	-	-	-	-	-	-	-	-	-	-	-	-
E6	Propyl acetate	-	-	-	-	-	25.81	65.47	132.99	303.55	186.34	-	-	-	-	-	-	-	-	-	-	-	-	-	-	-	-	-	-
E8	Ethyl butyrate	38.55	35.54	112.99	455.98	756.20	214.48	100.20	286.52	754.03	1039.17	-	-	-	-	-	-	-	-	0.00	1993.95	1226.82	197.10	1137.98	556.76	-	-	-	-
E9	Propyl propionate	-	-	-	-	-	43.67	30.76	57.33	451.95	567.35	-	-	-	-	-	-	-	-	-	-	-	-	-	-	-	-	-	-
E10	Butyl acetate	67.48	161.95	396.91	543.18	237.96	147.31	415.95	641.33	561.48	196.47	50.83	82.34	567.00	814.40	384.77	637.87	1051.00	860.98	-	-	-	-	-	-	-	-	-	-
E11	Ethyl 2-methylbutyrate	17.19	37.85	271.74	951.71	1271.18	118.82	80.29	296.45	1091.83	1960.53	-	-	-	-	-	-	-	-	0.00	932.82	248.81	226.38	1353.97	532.52	-	-	-	-
E12	2-Methylbutyl acetate	741.03	1417.92	1239.70	1321.73	584.89	1019.93	1289.32	1446.01	1416.43	1210.29	90.82	115.60	415.28	860.63	-	-	-	-	-	-	-	-	-	-	-	-	-	-
E13	Propyl butyrate	-	-	-	-	-	175.90	343.54	449.17	821.45	766.27	-	-	-	-	-	-	-	-	-	-	-	-	-	-	-	-	-	-
E14	Ethyl valerate	-	-	-	-	-	0.00	0.00	0.00	0.00	152.63	-	-	-	-	-	-	-	-	-	-	-	-	-	-	-	-	-	-
E20	Propyl 2-methylbutyrate	27.73	127.63	542.48	900.11	930.05	395.56	438.63	635.32	1306.63	1535.47	-	-	-	24.10	-	-	-	27.61	-	-	-	-	-	-	-	-	-	-
E23	Ethyl caproate	-	-	-	-	-	117.10	116.88	242.77	671.75	1224.89	-	-	-	-	-	-	-	-	-	-	-	-	-	-	-	-	-	-
E26	Hexyl acetate	170.14	354.93	635.30	746.78	191.74	508.78	1119.58	1554.79	1223.95	338.34	192.9097599	289.0911648	834.9625139	1015.268948	783.8779556	1148.960416	1585.754511	1280.6037	-	-	-	-	-	-	-	-	-	-
E27	2-Methyl-butanoic acid butyl ester	0.00	215.07	161.81	177.46	117.10	184.17	201.94	316.23	276.51	134.21	-	-	-	-	-	-	-	-	-	-	-	-	-	-	-	-	-	-
E42	Butyl hexanoate	144.99	239.25	136.27	175.68	167.15	-	-	-	-	-	112.73	168.96	330.90	303.41	195.93	318.45	223.11	114.58	-	-	-	-	-	-	-	-	-	-
E45	Hexyl 2-methylbutyrate	78.23	201.43	216.40	183.30	137.53	-	-	-	-	-	-	-	-	-	-	-	-	-	-	-	-	-	-	-	-	-	-	-
E52	1-Methylheptyl acetate	0.00	0.00	24.40	0.00	39.15	-	-	-	-	-	-	-	-	-	-	-	-	-	-	-	-	-	-	-	-	-	-	-

A3	Hexanal	-	-	-	-	-	83.11	19.99	215.18	0.00	0.00	0.00	0.00	0.00	101.74	-	-	-	-	-	-	-	-	-	-	48.21	546.14	-	-
A8	(E)-2-Octenal	-	-	-	-	-	188.37	94.67	95.97	95.54	92.46	-	-	-	-	-	-	-	-	-	-	-	0.00	0.00	121.33	-	-	-	-
B1	Ethanol	-	-	-	-	-	-	-	-	-	-	-	-	-	-	-	-	-	-	0.00	131.24	468.60	0.00	252.49	241.48	0.00	268.11	0.00	362.25
B3	1-Butanol	-	-	-	-	-	-	-	-	-	-	0.00	42.99	718.54	934.60	351.59	593.05	995.34	882.27	-	-	-	-	-	-	-	-	-	-
B5	2-Methyl-1-butanol	443.64	935.51	570.02	500.65	312.93	350.06	299.13	266.43	364.91	376.84	0.00	37.64	204.72	482.73	-	-	-	-	-	-	-	-	-	-	23.60	823.65	-	-
B9	Hexyl alcohol	-	-	-	-	-	-	-	-	-	-	0	465.7765867	1047.353577	1519.462741	922.1098119	1172.822929	1597.161149	1371.687391	175.11	855.30	388.95	798.28	666.89	450.49	0.00	507.81	302.21	0.00
B13	1,3-Octanediol	-	-	-	-	-	-	-	-	-	-	0.00	0.00	0.00	101.74	-	-	-	-	-	-	-	-	-	-	-	-	-	-
C4	Geranyl acetone	-	-	-	-	-	-	-	-	-	-	30.10	0.00	0.00	0.00	-	-	-	-	-	-	-	-	-	-	-	-	-	-
C5	β-Ionone	-	-	-	-	-	-	-	-	-	-	-	-	-	-	197.23	66.95	59.89	11.65	-	-	-	-	-	-	-	-	-	-
D1	2-Methyl butyric acid	50.84	291.07	484.37	679.82	163.37	-	-	-	-	-	-	-	-	-	-	-	-	-	-	-	-	-	-	-	-	-	-	-
T3	Estragole	17.56	35.01	123.18	117.98	63.03	0.00	159.06	174.51	135.38	68.18	-	-	-	-	-	-	-	-	-	-	-	-	-	-	-	-	-	-

-: no significant difference.

Table S4. The OAV of four apple cultivars in the first year.

Code	Compound	Aroma Description	Odor threshold（μg/kg）	Starking in skin					Starking in pulp					Golden Delicious in skin				Golden Delicious in pulp				Binzi in skin			Binzi in pulp			Xianggguo in skin		Xianggguo in pulp	
				S1	S2	S3	S4	S5	S1	S2	S3	S4	S5	S1	S2	S3	S4	S1	S2	S3	S4	S1	S2	S3	S1	S2	S3	S1	S2	S1	S2
E25	Methyl 2-methylbutyrate	Apple, fruit, green apple, strawberry	0.25	1388.35	0.00	1679.36	1810.44	7867.18	–	–	–	–	–	–	–	–	–	–	–	–	–	–	–	–	–	–	–	–	–	–	–
E7	Isobutyl acetate	Fruit, apple, banana, sweet	66	–	–	–	–	–	0.00	0.00	0.92	1.44	1.44	–	–	–	–	–	–	–	–	–	–	–	–	–	–	–	–	–	–
E8	Ethyl butyrate	Pineapple, fruity，apple	1	36.09	37.01	378.47	1041.48	982.00	38.55	35.54	112.99	455.98	756.20	–	–	–	–	–	–	–	–	0.00	3439.60	1727.43	0.00	1993.95	1226.82	–	–	–	–

E9	Propyl propionate	Apple, banana, pineapple	57	–	–	–	–	–	0.00	0.00	0.72	2.60	4.21	–	–	–	–	–	–	–	–	–	–	–	–	–	–	–	–	–	–
E10	Butyl acetate	Apple, banana	66	0.00	0.31	0.00	0.00	0.00	1.02	2.45	6.01	8.23	3.61	0.49	0.64	4.91	2.98	0.77	1.25	8.59	12.34	–	–	–	–	–	–	–	–	–	–
E11	Ethyl 2-methylbutyrate	Apple, ester, green Apple, kiwi, strawberry	0.1	332.13	562.86	9268.73	21121.72	23351.61	171.95	378.50	2717.36	1957.12	12711.84	–	–	–	–	–	–	–	–	0.00	15469.63	3837.02	0.00	9328.17	2488.15	608.27	8740.38	151.44	1036.50
E12	2-Methylbutyl acetate	Apple, banana, pear	5	154.18	214.20	204.46	125.66	8.45	148.21	283.58	247.94	264.35	116.98	17.36	13.88	74.90	124.98	18.16	23.12	83.06	172.13	–	–	–	–	–	–	0.00	0.00	2.06	0.00
E13	Propyl butyrate	Pineapple, solvent	18	1.50	2.23	27.60	54.95	36.01	0.67	1.39	9.59	21.31	19.22	–	–	–	–	–	–	–	–	–	–	–	–	–	–	–	–	–	–
E18	Methyl hexanoate	Apple, fruity, green, sweet, unripe	70	0.00	0.00	15.32	16.00	19.55	0.00	0.00	0.00	0.00	1.82	4.49	6.09	11.15	10.30	–	–	–	–	0.00	1.29	1.51	0.00	0.00	0.46	–	–	–	–
E22	Butyl butyrate	Banana	100	1.23	1.98	6.10	6.41	3.75	0.29	0.48	0.74	0.85	0.73	0.59	1.23	6.45	4.56	0.33	0.31	1.37	1.35	–	–	–	0.00	0.21	0.00	–	–	–	–
E23	Ethyl caproate	Apple peel, fruit	1	153.26	97.61	249.09	717.28	861.53	0.00	39.30	78.16	318.14	480.31	–	–	–	–	–	–	–	–	0.00	1167.97	782.60	0.00	325.01	260.21	0.00	83.08	–	–
E26	Hexyl acetate	Apple, banana, grass	2	227.07	197.35	92.73	0.00	0.00	85.07	177.47	317.65	373.39	95.87	164.92	266.55	532.83	140.10	96.45	144.55	417.48	507.63	–	–	–	–	–	–	–	–	–	–
E27	2-Methyl-butanoic acid butyl ester	Banana	17	0.00	52.45	111.92	80.33	55.26	0.00	12.65	9.52	10.44	6.89	0.00	0.00	28.17	55.32	0.00	0.00	5.64	11.22	–	–	–	–	–	–	–	–	–	–
E34	Hexyl propionate	Sweet, fruit	8	8.35	9.67	16.34	13.71	5.08	0.00	2.96	3.39	4.12	3.26	2.08	2.38	9.66	10.15	–	–	–	–	–	–	–	–	–	–	–	–	–	–
E36	Methyl octanoate	Fruit, orange, wax, wine	200	0.00	0.51	0.88	1.94	2.17	–	–	–	–	–	0.45	0.62	2.85	1.86	–	–	–	–	–	–	–	–	–	–	–	–	–	–
E42	Butyl hexanoate	Fruit, grass, green	700	0.84	1.27	1.92	2.58	2.12	0.21	0.34	0.19	0.25	0.24	0.27	0.42	2.11	1.72	0.16	0.24	0.47	0.43	0.00	0.23	0.00	–	–	–	1.10	0.61	–	–
E43	Ethyl caprylate	Apple, fruity, sweet	5	0.00	0.00	0.00	36.14	53.39	–	–	–	–	–	–	–	–	–	–	–	–	–	0.00	14.80	15.07	–	–	–	0.00	22.06	–	–
E45	Hexyl 2-methylbutyrate	Fruity, green, apple	6	103.91	286.63	527.09	294.90	254.87	13.04	33.57	36.07	30.55	22.92	12.97	24.97	183.89	242.33	6.57	11.53	31.48	34.43	0.00	19.27	0.00	–	–	–	74.47	41.42	–	–
E48	Hexanoic acid, pentyl ester	Banana, pineapple	4.38	11.92	25.29	37.37	45.39	39.58	–	–	–	–	–	0.00	0.00	16.84	19.22	–	–	–	–	–	–	–	–	–	–	–	–	–	–

E51	Hexyl hexanoate	Fresh fruit	10	0.00	0.00	116.14	105.23	122.60	–	–	–	–	–	0.00	0.00	124.75	92.33	–	–	–	–	0.00	12.93	0.00	–	–	–	–	–	–	–
E55	Hexyl caprylate	Vegetable, fruit	2	–	–	–	–	–	–	–	–	–	–	0.00	0.00	30.60	25.92	–	–	–	–	–	–	–	–	–	–	–	–	–	–
A1	2-Methyl butyraldehyde	Almond, cocoa, fermented, hazelnut, malt	1	39.60	50.52	101.76	109.60	160.10	–	–	–	–	–	0.00	0.00	36.57	75.64	–	–	–	–	–	–	–	–	–	–	–	–	–	–
A2	Pentanal	Banana, grass, lower aldehyde	12	4.93	5.14	0.00	7.03	7.01	–	–	–	–	–	7.32	6.04	7.32	8.63	–	–	–	–	–	–	–	–	–	–	–	–	–	–
A3	Hexanal	Grass, green, leaves, vinous	4	282.97	305.19	402.54	327.78	359.45	10.64	10.99	4.54	3.84	8.10	320.70	257.55	407.84	0.00	–	–	–	–	23.32	42.80	113.58	3.87	0.00	8.49	392.64	257.22	12.05	136.53
A4	Trans-2-hexenal	Almond, bitter, green	17	0.00	1.74	0.00	1.13	1.39	–	–	–	–	–	–	–	–	–	–	–	–	–	–	–	–	–	–	–	–	–	–	–
A5	Heptaldehyde	Fresh, herbal	3	–	–	–	–	–	–	–	–	–	–	0.00	0.00	116.18	131.51	–	–	–	–	–	–	–	–	–	–	–	–	–	–
A6	(Z)-Hept-2-en	Fatty, fruity, green	13	0.00	0.00	14.81	18.57	9.56	–	–	–	–	–	0.00	23.30	0.00	7.54	–	–	–	–	10.48	37.04	22.05	–	–	–	16.82	0.00	–	–
A8	(E)-2-Octenal	Sweet, green, fatty, herbal, cucumber	90	2.12	3.21	5.14	4.59	3.26	0.45	0.63	0.29	0.24	0.21	2.17	2.95	4.25	5.45	–	–	–	–	0.66	1.24	1.19	0.00	0.20	0.26	3.70	3.04	0.54	0.67
A10	1-Nonanal	Aldehyde, citrus, fatty, floral, green	1	–	–	–	–	–	–	–	–	–	–	166.74	192.27	335.04	181.99	–	–	–	–	26.49	0.00	84.79	–	–	–	151.52	116.24	–	–
A12	Trans-2-decenal	Fatty, mushroom	10	–	–	–	–	–	–	–	–	–	–	–	–	–	–	–	–	–	–	0.00	3.85	0.00	–	–	–	–	–	–	–
A13	Citral	Lemon	32	0.00	1.33	2.45	1.91	0.00	–	–	–	–	–	0.00	0.00	1.10	0.00	–	–	–	–	–	–	–	–	–	–	0.00	1.75	0.00	0.00
B1	Citral	Alcoholic, strong	620	–	–	–	–	–	–	–	–	–	–	–	–	–	–	–	–	–	–	0.00	0.38	1.57	0.00	0.21	0.76	0.00	0.81	0.00	0.43
B3	2-Undecenal	Floral, fragrant, fruity, sweet	500	0.00	0.00	0.76	0.91	0.83	–	–	–	–	–	0.00	0.03	2.83	3.25	0.00	0.09	1.44	1.87	–	–	–	–	–	–	–	–	–	–
B5	Ethanol	Alcoholic, banana, iodoform, vinous	250	6.56	5.86	7.54	5.19	3.05	1.77	3.74	2.28	2.00	1.25	0.00	0.00	1.72	3.61	0.00	0.15	0.82	1.93	0.00	1.55	0.61	0.00	0.95	0.00	0.61	2.09	0.09	3.29
B9	Hexyl alcohol	Resin, flower, green	500	–	–	–	–	–	–	–	–	–	–	–	–	–	–	0.00	0.93	2.09	3.04	0.76	2.97	3.54	0.35	1.71	0.78	0.00	0.00	0.00	1.02
B12	1-Octen-3-ol	Fatty, fruity, grass, mushroom, sweet	10	12.95	15.39	22.90	25.50	34.04	–	–	–	–	–	–	–	–	–	–	–	–	–	–	–	–	4.24	3.85	2.79	22.68	0.00	7.43	10.30

C3	1-Hepten-3-one	Citrus pepper, mushroom, rubber	50	14.90	22.74	64.69	59.63	82.88	0.00	0.00	0.00	0.00	2.72	4.88	7.53	29.19	56.20	–	–	–	–	4.58	73.49	19.47	0.00	0.00	1.74	30.41	52.83	0.00	2.79
C4	Geranyl acetone	Fruit	60	–	–	–	–	–	–	–	–	–	–	0.00	0.00	0.00	0.70	0.50	0.00	0.00	0.00	–	–	–	–	–	–	1.69	0.95	0.47	0.79
C5	β-Ionone	Seaweed, violet, flower, raspberry	7	–	–	–	–	–	0.00	1.33	2.17	2.46	4.38	–	–	–	–	1.57	1.73	1.42	1.55	–	–	–	–	–	–	–	–	–	–
C6	β-Damascenone	Apple, rose, honey	0.05	–	–	–	–	–	691.43	627.39	769.58	825.32	573.62	–	–	–	–	795.87	677.68	921.84	873.31	–	–	–	241.95	0.00	218.93	–	–	775.81	1574.64
D1	2-Methyl butyric acid	Pungent, cheese, fruity	5.8	166.19	124.66	606.44	0.00	0.00	8.77	50.18	83.51	117.21	28.17	–	–	–	–	–	–	–	–	0.00	13.58	0.00	–	–	–	–	–	–	–
T21	Linalool	Flower, lavender	6	–	–	–	–	–	–	–	–	–	–	–	–	–	–	–	–	–	–	–	–	–	–	–	–	0.00	21.47	–	–

–: not detected.

Table S5 The OAV of four apple cultivars in the second year.

Code	Compound	Aroma Description	Odor threshold（μg/kg）	Starking in skin					Starking in pulp					Golden Delicious in skin				Golden Delicious in pulp				Binzi in skin			Binzi in pulp			Xiangguo in skin		Xiangguo in pulp	
				S1	S2	S3	S4	S5	S1	S2	S3	S4	S5	S1	S2	S3	S4	S1	S2	S3	S4	S1	S2	S3	S1	S2	S3	S1	S2	S1	S2
E5	Methyl 2-methylbutyrate	Apple, fruit, green apple, strawberry	0.25	460.03	218.48	277.10	281.49	510.60	–	–	–	–	–	–	–	–	–	–	–	–	–	–	–	–	–	–	–	–	–	–	–
E7	Isobutyl acetate	Fruit, apple, banana, sweet	66	–	–	–	–	–	0.00	0.85	0.98	1.01	1.16	–	–	–	–	–	–	–	–	–	–	–	–	–	–	–	–	–	–
E8	Ethyl butyrate	Pineapple, fruity，apple	1	71.39	64.60	165.20	662.96	1038.81	214.48	100.20	286.52	754.03	1039.17	–	–	–	–	–	–	–	–	290.77	330.79	153.85	197.10	1137.98	556.76	–	–	–	–
E9	Propyl propionate	Apple, banana, pineapple	57	–	–	–	–	–	0.77	0.54	1.01	7.93	9.95	–	–	–	–	–	–	–	–	–	–	–	–	–	–	–	–	–	–
E10	Butyl acetate	Apple, Banana	66	0.71	0.81	0.77	0.49	0.00	2.23	6.30	9.72	8.51	2.98	4.81	6.13	6.44	6.57	5.83	9.66	15.92	13.05	–	–	–	–	–	–	–	–	–	–
E11	Ethyl 2-methylbutyrate	Apple, ester, green apple, kiwi, strawberry	0.1	1439.73	873.55	2077.79	9931.57	18677.28	1188.18	802.89	2964.47	10918.33	19605.28	–	–	–	–	–	–	–	–	2277.63	12651.66	7438.09	2263.78	13539.65	5325.16	0.00	514.13	0.00	233.99
E12	2-Methylbutyl acetate	Apple, banana, pear	5	160.23	159.69	157.71	158.59	58.63	203.99	257.86	289.20	283.29	242.06	43.71	59.84	57.50	78.58	46.71	74.34	101.96	104.38	–	–	–	–	–	–	0.00	0.00	0.00	3.61

E13	Propyl butyrate	Pineapple, solvent	18	7.59	11.07	17.30	41.84	47.84	9.77	19.09	24.95	45.64	42.57	–	–	–	–	–	–	–	–	–	–	–	–	–	–	–	–	–	–
E18	Methyl hexanoate	Apple, fruity, green, sweet, unripe	70	3.63	2.80	5.33	5.48	6.71	0.00	0.00	0.00	0.00	1.20	0.00	3.49	4.96	0.00	–	–	–	–	1.62	2.83	3.67	0.00	0.30	0.33	–	–	–	–
E22	Butyl butyrate	Banana	100	4.20	7.13	8.05	8.68	6.37	0.76	1.70	2.24	1.57	0.00	4.40	7.79	8.62	6.31	0.97	1.65	2.75	1.22	–	–	–	0.18	0.00	0.00	–	–	–	–
E23	Ethyl caproate	Apple peel, fruit	1	118.34	156.73	223.12	900.67	1644.81	117.10	116.88	242.77	671.75	1224.89	–	–	–	–	–	–	–	–	132.75	0.00	184.93	44.60	410.23	184.34	0.00	27.08	–	–
E26	Hexyl acetate	Apple, banana, grass	2	265.20	331.59	431.17	246.19	0.00	254.39	559.79	777.39	611.98	169.17	615.45	623.20	644.53	606.25	391.94	574.48	792.88	640.30	–	–	–	–	–	–	–	–	–	–
E27	2-Methyl-butanoic acid butyl ester	Banana	17	55.97	65.65	86.90	80.98	53.28	10.83	11.88	18.60	16.27	7.89	18.28	29.47	38.25	40.87	0.00	4.96	9.32	6.04	–	–	–	–	–	–	–	–	–	–
E34	Hexyl propionate	Sweet, fruit	8	50.25	35.72	56.95	79.01	47.65	0.00	2.90	4.69	8.95	5.56	29.33	54.20	56.77	57.84	–	–	–	–	–	–	–	–	–	–	–	–	–	–
E36	Methyl octanoate	Fruit, orange, wax, wine	200	0.83	0.82	1.42	1.03	0.99	–	–	–	–	–	0.59	0.75	1.20	0.54	–	–	–	–	–	–	–	–	–	–	–	–	–	–
E42	Butyl hexanoate	Fruit, grass, green	700	2.25	2.52	2.91	3.07	2.17	0.09	0.23	0.25	0.18	0.06	1.73	2.70	2.63	2.22	0.23	0.38	0.78	0.17	0.13	0.12	0.00	–	–	–	0.18	0.00	–	–
E43	Ethyl caprylate	Apple, fruity, sweet	5	0.00	0.00	0.00	31.80	62.98	–	–	–	–	–	–	–	–	–	–	–	–	–	20.67	26.85	23.52	–	–	–	0.00	6.73	–	–
E45	Hexyl 2-methylbutyrate	Fruity, green, apple	6	369.06	275.56	382.41	383.97	356.76	11.75	22.97	25.42	22.72	9.96	174.48	202.30	173.20	179.01	10.73	21.66	36.07	12.41	44.51	32.92	1.75	–	–	–	16.46	0.00	–	–
E48	Hexanoic acid, pentyl ester	Banana, pineapple	4.38	87.84	81.09	84.44	102.33	87.65	–	–	–	–	–	26.38	53.36	38.93	31.62	–	–	–	–	–	–	–	–	–	–	–	–	–	–
E51	Hexyl hexanoate	Fresh fruit	10	149.92	118.20	140.16	138.86	98.82	–	–	–	–	–	103.05	136.83	114.81	113.60	–	–	–	–	51.33	0.00	0.88	–	–	–	–	–	–	–
E55	Hexyl caprylate	Vegetable, fruit	2	–	–	–	–	–	–	–	–	–	–	34.15	45.16	37.38	30.83	–	–	–	–	–	–	–	–	–	–	–	–	–	–
A1	2-Methylbutyraldehyde	Almond, cocoa, fermented, hazelnut, malt	1	13.17	16.60	19.66	20.78	16.50	–	–	–	–	–	0.00	0.00	9.31	12.90	–	–	–	–	–	–	–	–	–	–	–	–	–	–
A2	Pentanal	Banana, grass, lower aldehyde	12	1.12	0.83	0.00	1.18	0.00	–	–	–	–	–	1.37	1.25	2.52	2.56	–	–	–	–	–	–	–	–	–	–	–	–	–	–
A3	Hexanal	Grass, green, leaves, vinous	4	60.78	38.43	46.56	77.94	85.68	20.78	5.00	53.80	0.00	0.00	34.87	59.12	118.12	140.66	–	–	–	–	14.33	22.15	57.01	3.33	0.00	0.00	26.82	31.21	0.00	6.41
A4	Trans-2-hexenal	Almond, bitter, green	17	5.61	0.00	0.00	0.00	4.74	–	–	–	–	–	–	–	–	–	–	–	–	–	–	–	–	–	–	–	–	–	–	–
A5	Heptaldehyde	Fresh, herbal	3	–	–	–	–	–	–	–	–	–	–	40.03	0.00	0.00	57.22	–	–	–	–	–	–	–	–	–	–	–	–	–	–

A6	(Z)-Hept-2-en	Fatty, fruity, green	13	40.23	50.36	50.73	52.70	47.31	–	–	–	–	–	31.51	19.93	27.23	52.55	–	–	–	–	6.06	3.80	27.54	–	–	–	9.37	1.42	–	–
A8	(E)-2-Octenal	Sweet, green, fatty, herbal, cucumber	90	4.06	3.94	3.87	3.66	2.88	2.09	1.05	1.07	1.06	1.03	2.96	2.61	3.13	3.35	–	–	–	–	3.23	2.56	4.04	0.00	0.00	1.35	3.35	2.64	0.00	0.50
A10	1-Nonanal	Aldehyde, citrus, fatty, floral, green	1	–	–	–	–	–	–	–	–	–	–	193.56	205.30	152.52	111.15	–	–	–	–	104.78	109.19	221.41	–	–	–	37.64	30.85	–	–
A12	Trans-2-decenal	Fatty, mushroom	10	–	–	–	–	–	–	–	–	–	–	–	–	–	–	–	–	–	–	12.23	7.01	8.34	–	–	–	–	–	–	–
A13	Citral	Lemon	32	1.46	1.65	1.78	32.06	2.77	–	–	–	–	–	0.00	1.49	0.88	0.66	–	–	–	–	–	–	–	–	–	0.00	0.00	0.50	0.00	0.00
B1	Citral	Alcoholic, strong	620	–	–	–	–	–	–	–	–	–	–	–	–	–	–	–	–	–	–	0.00	0.28	1.01	0.00	0.41	0.39	0.00	0.38	0.00	0.58
B3	2-Undecenal	Floral, fragrant, fruity, sweet	500	0.15	0.19	0.40	0.65	0.80	–	–	–	–	–	0.33	0.90	1.67	2.38	0.70	1.19	1.99	1.76	–	–	–	–	–	–	–	–	–	–
B5	Ethanol	Alcoholic, banana, iodoform, vinous	250	1.89	1.71	2.06	2.68	3.10	1.40	1.20	1.07	1.46	1.51	0.24	0.51	0.84	1.62	0.40	0.75	1.00	1.23	0.64	1.34	3.25	0.72	1.44	1.36	0.00	0.17	0.07	0.06
B9	Hexyl alcohol	Resin, flower, green	500	–	–	–	–	–	–	–	–	–	–	–	–	–	–	1.84	2.35	3.19	2.74	1.68	1.08	1.59	1.60	1.33	0.90	0.00	0.00	0.60	0.00
B12	1-Octen-3-ol	Fatty, fruity, grass, mushroom, sweet	10	25.20	18.76	21.86	17.74	0.00	–	–	–	–	–	–	–	–	–	–	–	–	–	–	–	–	18.40	16.43	27.56	7.29	4.07	3.43	0.00
C3	1-Hepten-3-one	Citrus Pepper, mushroom, rubber	50	33.56	37.25	42.88	41.07	23.03	2.20	1.85	0.00	0.00	0.00	32.21	31.25	38.80	22.56	–	–	–	–	20.62	28.62	20.51	0.84	1.39	1.16	27.19	33.50	0.51	1.45
C4	Geranyl acetone	Fruit	60	–	–	–	–	–	–	–	–	–	–	1.21	0.00	0.00	0.00	0.49	0.39	0.00	0.00	–	–	–	–	–	0.00	0.00	0.68	0.40	0.00
C5	β-Ionone	Seaweed, violet, flower, raspberry	7	–	–	–	–	–	0.00	0.00	0.00	1.13	0.99	–	–	–	–	28.18	9.56	8.56	1.66	–	–	–	–	–	–	–	–	–	–
C6	β-Damascenone	Apple, rose, honey	0.05	–	–	–	–	–	0.00	0.00	0.00	0.00	308.92	–	–	–	–	470.40	690.91	596.35	639.12	–	–	–	177.36	186.83	0.00	–	–	156.60	180.68
D1	2-Methyl butyric acid	Pungent, cheese, fruity	5.8	149.06	312.69	377.54	220.23	124.08	53.44	60.61	57.12	58.88	56.22	–	–	–	–	–	–	–	–	37.27	19.62	0.00	–	–	–	–	–	–	–
T2	Linalool	Flower, lavender	6	–	–	–	–	–	–	–	–	–	–	–	–	–	–	–	–	–	–	–	–	–	–	–	–	10.35	15.84	–	–

–: not detected.
